# The characteristics and analysis of the complete chloroplast genome of *Hemerocallis* cultivar Small orange lamp 2019 (Asphodelaceae)

**DOI:** 10.1080/23802359.2024.2435904

**Published:** 2024-12-02

**Authors:** Xiaofei Zhang, Jiaming Yang, Xuwen Shang, Shengyue Chai, Lanling Jiang, Xiaolei Hou, Zhenting Wang, Lixin Lang

**Affiliations:** a Liaoning Academy of Agricultural Sciences, Institute of Flowers, Shenyang, China; b Shenyang Agricultural University, Shenyang, China; c China State Construction Corporation Limited, Shenyang, China

**Keywords:** Chloroplast genome, *Asphodelaceae*, *Hemerocallis* cultivar Small orange lamp, phylogenetic analysis

## Abstract

*Hemerocallis* cultivar Small orange lamp is a hybrid variety. Its whole chloroplast genome was 156,053 bp in size, consisting of 135 genes in total, including 89 mRNA genes, 38 tRNA genes, and 8 rRNA genes. The chloroplast genome contained a large single copy region (LSC 84,805 bp), a small single copy region (SSC 18,510 bp) and a pair of inverted repeats (IRa and IRb 26,369 bp). The overall GC content was 37.34%. Phylogenetic analysis showed that *Hemerocallis lilioasphodelus* and *H*. cultivar Small orange lamp are most closely related. This will provide valuable reference for further research on the genetic information of plants in the *Hemerocallis.*

## Introduction

*Hemerocallis* is known as the traditional mother’s flower in Chinese culture with a history of over 1000 years (Zhang et al. 2023). It is widely used in gardens as one of the world’s three major perennial flowers. According to reports, *Hemerocallis* has antioxidant, sedative, anti-inflammatory, hypnotic, antidepressant, antitumor, and neuroprotective effects (Jiang et al. 2024). *Hemerocallis* cultivar Small orange lamp is a new variety developed through hybrid breeding and registered in the American Daylily Society in 2019 (www.daylilies.org/DaylilyDB/detail.php?id=189419&name=Small%20Orange%20Lamp) by Liaoning Academy of Agricultural Sciences, China. It is a perennial herbaceous plant of the *Asphodelaceae* family, with orange red flowers and a group flowering period of more than 2 months. It can bloom for many years after being planted once and can withstand low temperatures of −30 °C. The germplasm resources of *Hemerocallis* are extremely abundant, but their classification has always been a hot topic of discussion (Chase et al. 2016). Until 2016, the APG IV classification system designated *Hemerocallis* as a subfamily of *Asphodelaceae*, namely the *Subfam. Hemerocallidoideae*, which includes the tribe *Trib. Hemerocallidae*, *Hemerocallis* L. In order to analyze the phylogenetic relationship of *Hemerocallis*, we sequenced the complete chloroplast genome of *H.* cultivar Small orange lamp by using Illumina sequencing techniques, and studied its genomic characteristics.

## Materials and methods

**Figure 1.**
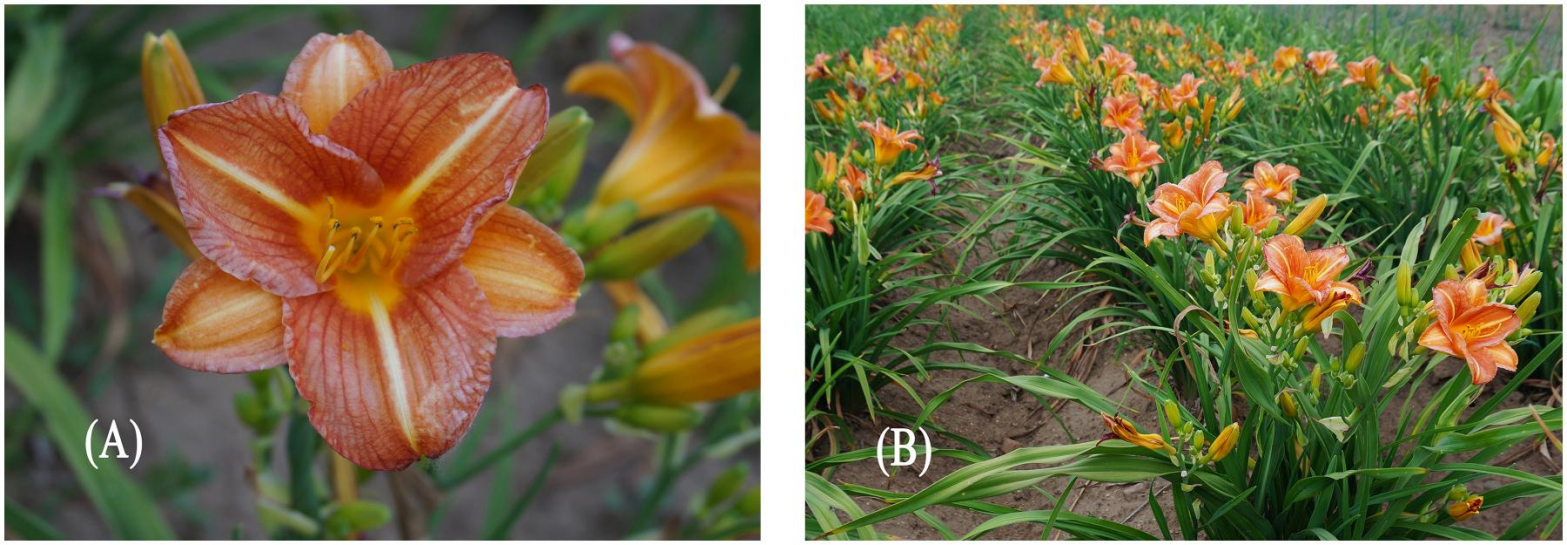
The photos of *Hemerocallis* cultivar Small orange lamp. (A) Flower, (B) Group landscape. Its flowers are orange red in color, with a plant height of 30∼40 centimeters. Both photos were taken by the author Zhang Xiaofei.

We collected the fresh leaf samples of *H.* cultivar Small orange lamp from the Germplasm Resource Garden of Institute of Floriculture, Liaoning Academy of Agricultural Sciences (41°49′14″N, 123°32′47″E), Shenyang city, Liaoning province, China (Figure 1). The voucher specimen assigned ASPH_HEM_FUL_01 is deposited at Institute of Floriculture of Liaoning Academy of Agricultural Sciences (Xiaofei Zhang and 1249308231@qq.com).

We used the improved CTAB method (Yang et al. 2014) to extract the complete chloroplast genome DNA, and sequenced on the Illumina NovaSeq 6000 sequencing platform, the sequencing reads paired-end (PE) is 150 bp. The high-quality reads obtained by filtering the raw data using fastp(version 0.20.0, https://github.com/OpenGene/fastp) software, filtering out reads with an average value less than Q5, and a series of quality controls are called clean data. The assembly of chloroplast genome is carried out using SPAdes software (Bankevich et al. 2012), and k-mers were set to 55, 87and 121. To improve the accuracy of annotation, we used two methods to annotate the chloroplast genome. Firstly, prodigal v2.6.3 (https://www.github.com/hyattpd/Prodigal) software was used to annotate the CDS of chloroplast, hmmer v3.1b2 (http://www.hmmer.org/) software was used to predict rRNA, and aragorn v1.2.38 (http://130.235.244.92/ARAGORN/) software was used to predict tRNA. Secondly, blast v2.6 (https://blast.ncbi.nlm.nih.gov/Blast.cgi) software was used to compare the assembled sequences, based on the published closely related species *Hemerocallis citrina* (MN872235.1) on NCBI. Then the differential genes between the two annotation results were manually checked to remove errors and redundancy, and the final annotation was obtained. CPGview (http://47.96.249.172:16085/cpgview/home) software was used to draw the chloroplast genome map, cis-splicing gene map and trans-splicing gene map of *H.* cultivar Small orange lamp. In addition, we also conducted some genomic structure studies, including RSCU (Relative Synonymous Codon Usage) analysis and cpSSR (the Simple Sequence Repeats of chloroplast genome) analysis. Perl script MISA v1.0 software was used to analysis of the cpSSR (Thiel et al. 2003).

To investigate the phylogenetic relationship between *H.* cultivar Small orange lamp and other plants in *Asphodelaceae*, 23 complete chloroplast genome sequences were downloaded from NCBI, including 22 *Asphodelaceae* plants and 1 *Iridaceae* plant, *Iris tectorum*(MT103435.1), as the out-groups. MEGA6 (Tamura et al. 2013) software was used to construct the phylogenetic trees by the Maximum Likelihood method with 1000 bootstrap replications based on the Tamura-Nei model (Tamura and Nei 1993). The analysis involved 23 whole plastome sequences. The tree with the highest log likelihood (-3829756.5559) is shown. The tree is drawn to scale, with branch lengths measured in the number of substitutions per site. Codon positions included were 1st + 2nd + 3rd + Noncoding. All positions containing gaps and missing data were eliminated. There were a total of 145361 positions in the final dataset.

## Results

**Figure 2.**
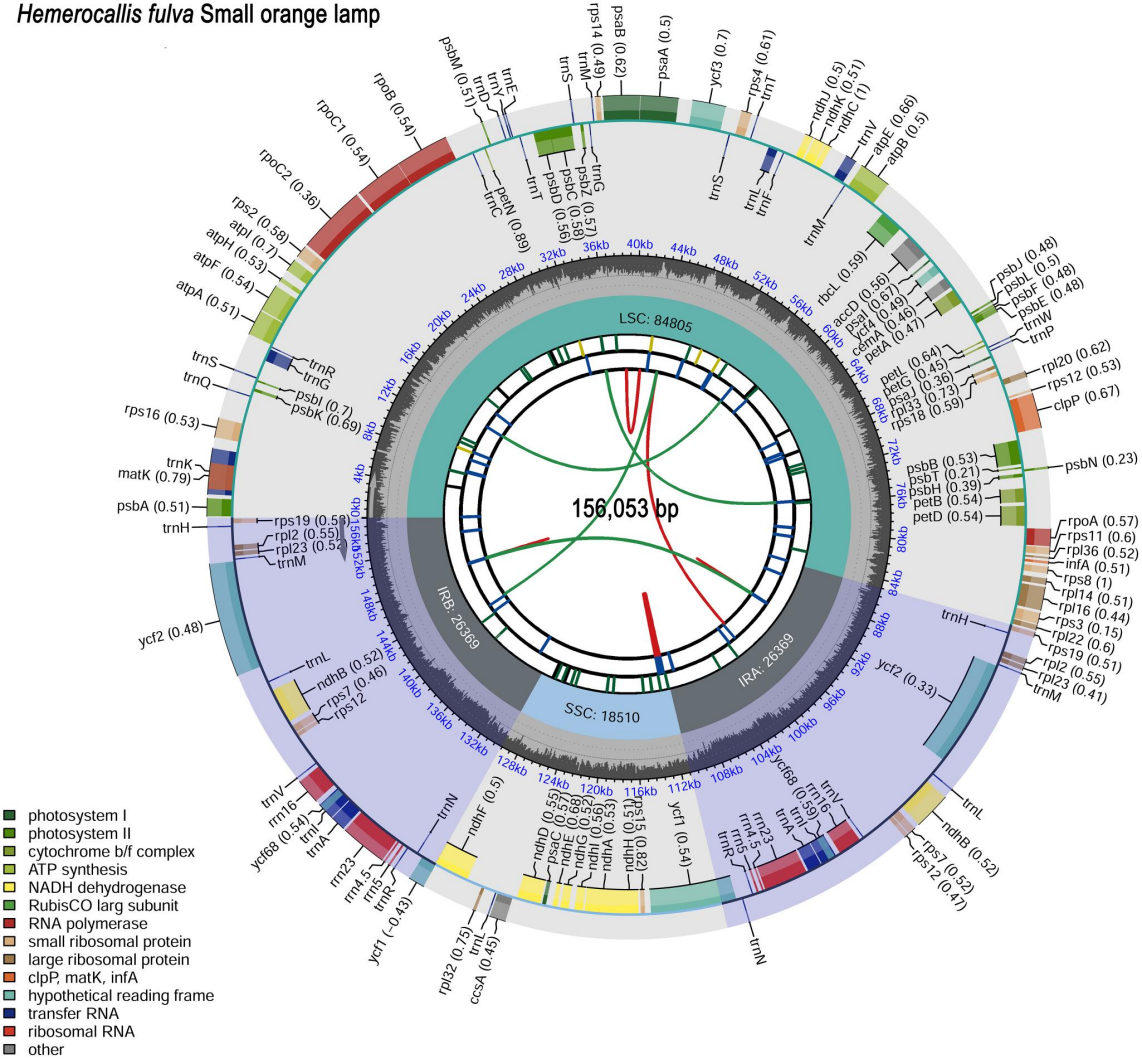
The chloroplast genome map of *Hemerocallis* cultivar Small orange lamp. The genome length markers are located in the middle of the image. The map contains six tracks, from the center outward, the first track shows the dispersed repeats. The second track shows the long tandem repeats as short blue bars. The third track shows the short tandem repeats or microsatellite sequences as short bars with different colors. The fourth track shows the regions of LSC, SSC, IRa and IRb. The fifth track shows the GC content. And the sixth track shows the genes names, the transcription directions for the genes drawn outside and inside the outer circle are counterclockwise and clockwise, respectively. The legend in different colors in the bottom left corner is used to distinguish genes with different functions.

The chloroplast genome of *H.* cultivar Small orange lamp has been deposited into the NCBI GenBank database with the accession number ON553913.1. This genome sequence has a total length of 156,053bp and a GC content of 37.34% (Figure 2). The average read mapping depths of the assembled genome were 1127.87× (Figure S3). It is a typical quadripartite structure, consisting of a large single copy region (LSC), a small single copy region (SSC), and a pair of inverted repeat regions (IRa and IRb), with lengths of 84,805 bp, 18,510 bp, and 26,369 bp, respectively. A total of 135 genes were annotated in the chloroplast genome, including 89 protein-coding genes, 38 tRNA genes, and 8 rRNA genes. 15 genes contain one intron, including *ndhA*, *ndhB*, *petB*, *petD*, *atpF*, *rpl16*, *rps12*, *rps16*, *rpoC1*, *trnA-UGC*, *trnG-UCC*, *trnI-GAU*, *trnK-UUU*, *trnL-UAA*, *trnV-UAC*, while 2 genes contain two introns, including *clpP* and *ycf3*. 11 cis-splicing genes including rps16, atpF, rpoC1, ycf3, clpP, petB, petD, rpl16, ndhA and ndhB (two copies) (Figure S1), and rps12 with three unique exons is a trans-spliced gene (Figure S2). 249 SSRs were detected in the whole chloroplast genome. There are a total of 33 high-frequency codons in the genome with RSCU≧1, with 13 and 16 high-frequency codons ending in A and U, accounting for 87.88% of the total. There are 3 high-frequency codons ending in G and 1 high-frequency codon ending in C, indicating that the chloroplast codon of *H.* cultivar Small orange lamp prefers to use A or U endings. The phylogenetic tree showed that all *Hemerocallis* species were classified as a monophyletic branch and indicated a close relationship among *Hemerocallis fulva* (MG914655.1), *Hemerocallis middendorffii* (OR260124.1), *Hemerocallis lilioasphodelus* (OR260110.1) and *H.* cultivar Small orange lamp (ON553913.1).

**Figure 3.**
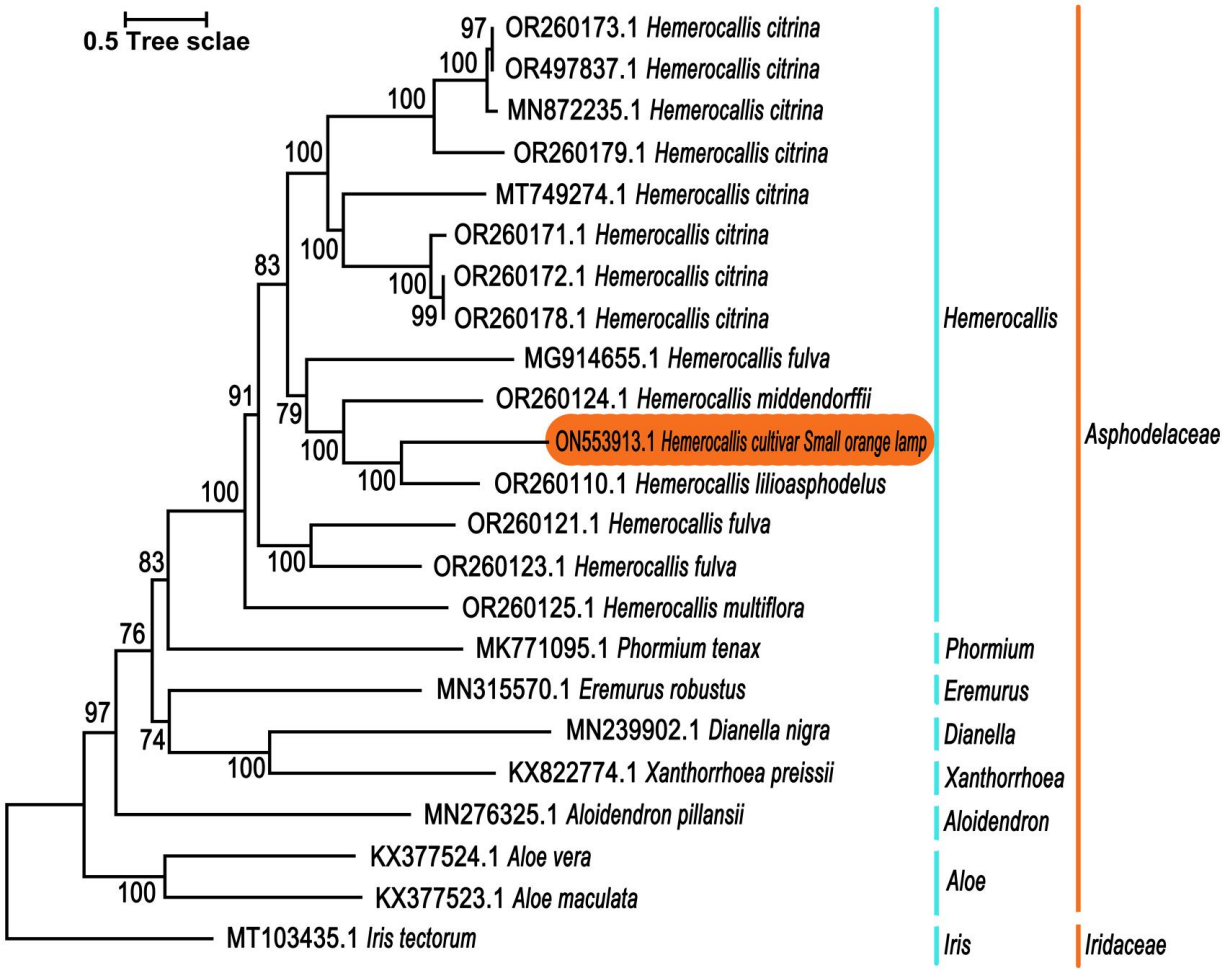
The phylogenetic tree of 23 plant species, including *Hemerocallis* cultivar Small orange lamp. 22 plant species belong to the family *Asphodelaceae*, and 1 plant species belongs to the family *Iridaceae*. All the above genome sequences were downloaded from NCBI GenBank. The following sequences were used: *H.* cultivar Small orange lamp ON553913.1 (Zhang. 2022), *H. citrina* OR260173.1 (Wu. 2023), *H. citrina* OR497837.1 (Jia, Wu, Z, et al. 2024), *H. citrina* MN872235.1 (Zhao and Liu. 2020), *H. citrina* OR260179.1 (Wu. 2023), *H. citrina* MT749274.1 (Wu and Li. 2022), *H. citrina* OR260171.1 (Wu. 2023), *H. citrina* OR260172.1 (Wu. 2023), *H. citrina* OR260178.1 (Wu. 2023), *Hemerocallis fulva* MG914655.1 (Lee and Nah. 2019), *H. fulva* OR260121.1 (Wu. 2023), *H. fulva* OR260123.1 (Wu. 2023), *Hemerocallis middendorffii* OR260124.1 (Wu. 2023), *Hemerocallis lilioasphodelus* OR260110.1 (Wu. 2023), *Hemerocallis multiflora* OR260125.1 (Wu. 2023), *Aloe vera* KX377524.1 (Lee and Yang. 2017), *Aloe maculata* KX377523.1 (Lee and Yang. 2017), *Aloidendron pillansii* MN276325.1 (Malakasi, Bellot, Dee, et al. 2019), *Eremurus robustus* MN315570.1 (Makhmudjanov, Yusupov, Abdullaev, et al. 2020), *Xanthorrhoea preissii* KX822774.1 (Park, Kim and Kim. 2017), *Phormium tenax* MK771095.1 (Smissen. 2021), *Dianella nigra* MN239902.1 (Smissen. 2021), and *Iris tectorum* MT103435.1 (Yu. 2021).

## Discussion and conclusion

This study employed the complete chloroplast genome of *H.* cultivar Small Orange Lamp, along with 22 other reported closely related species, for phylogenetic analysis. The research results show that *H. lilioasphodelus* and *H. cultivar* Small orange lamp are most closely related, we speculate that *H. lilioasphodelus* is the potential maternal parent of *H. cultivar* Small orange lamp, but further research is needed to confirm this. The study also found that *H. fulva* is the non-monophyly, which could be due to the long horticultural history for the genus. At present, there are over 100000 varieties of *Hemerocallis* spp. registered in the American Daylily Society (Misiukevičius et al. 2023). The phylogenetic tree showed that all *H. citrina* species were classified as a monophyletic branch, this indicates that the *H. citrina* has a relatively stable heritability, which may also be related to its unique nighttime flowering. Li et al. found that there is gene exchange between *H. citrina* and *H. fulva*, but this research did not confirm it, which may be related to the small sample size in this research (Li et al. 2020). This study sequenced, assembled, and annotated the entire genome of *H.* cultivar Small orange lamp, and analyzed the phylogenetic relationships of the family *Asphodelaceae*. It also provides important information for further research on genetic diversity, phylogenetic relationships, and resource conservation of *Hemerocallis*.

## Supplementary Material

Supplemental Material

Supplemental Material

Supplemental Material

## Data Availability

The genome sequence data that support the findings of this study are openly available in GenBank of NCBI at https://www.ncbi.nlm.nih.gov/nuccore/ON553913.1 under the accession No. ON553913.1. The associated Bio-Project, SRA and Bio-Sample numbers are PRJNA839863, SRR19360591 and SAMN28556749, respectively.

## References

[CIT0001] Bankevich A, Nurk S, Antipov D, Gurevich AA, Dvorkin M, Kulikov AS, Lesin VM, Nikolenko SI, Pham S, Prjibelski AD, et al. 2012. SPAdes: a new genome assembly algorithm and its applications to single-cell sequencing. J Comput Biol. 19(5):455–477. doi:10.1089/cmb.2012.0021. 22506599 PMC3342519

[CIT0002] Chase MW, Christenhusz MJM, Fay MF, et al. 2016. An update of the Angiosperm Phylogeny Group classification for the orders and families of flowering plants: APG IV. Bot J Linn Soc. 181(1):1–20. doi:10.1111/boj.12385.

[CIT0003] Jiang N, Zhang Y, Yao C, Chen F, Liu Y, Chen Y, Wang Y, Choudhary MI, Liu X. 2024. Hemerocallis citrina Baroni ameliorates chronic sleep deprivation-induced cognitive deficits and depressive-like behaviours in mice. Life Sci Space Res (Amst). 40:35–43. doi:10.1016/j.lssr.2023.04.001. 38245346

[CIT0004] Li S, Ji F, Hou F, Cui H, Shi Q, Xing G, Weng Y, Kang X. 2020. Characterization of Hemerocallis citrina transcriptome and development of EST-SSR markers for evaluation of genetic diversity and population structure of Hemerocallis collection. Front Plant Sci. 11:686. doi:10.3389/fpls.2020.00686. 32595657 PMC7300269

[CIT0005] Misiukevičius E, Mažeikienė I, Gossard J, Starkus A, Stanys V. 2023. Transcriptome analysis of diploid and autotetraploid Hemerocallis response to drought stress. Horticulturae. 9(11):1194. doi:10.3390/horticulturae9111194.

[CIT0007] Tamura K, Stecher G, Peterson D, Filipski A, Kumar S. 2013. MEGA6: molecular evolutionary genetics analysis version 6.0. Mol Biol Evol. 30(12):2725–2729. doi:10.1093/molbev/mst197. 24132122 PMC3840312

[CIT0008] Tamura K, Nei M. 1993. Estimation of the number of nucleotide substitutions in the control region of mitochondrial DNA in humans and chimpanzees. Mol Biol Evol. 10(3):512–526. doi:10.1093/oxfordjournals.molbev.a040023. 8336541

[CIT0009] Thiel T, Michalek W, Varshney RK, Graner A. 2003. Exploiting EST databases for the development and characterization of gene-derived SSR-markers in barley (Hordeum vulgare L.). Theor Appl Genet. 106(3):411–422. doi:10.1007/s00122-002-1031-0. 12589540

[CIT0010] Yang JB, Li DZ, Li HT. 2014. Highly effective sequencing whole chloroplast genomes of angiosperms by nine novel universal primer pairs. Mol Ecol Resour. 14(5):1024–1031. doi:10.1111/1755-0998.12251. 24620934

[CIT0011] Zhang L, Zhou L, Meng J, Wu S, Liu S, Yang N, Tian F, Yu X. 2023. Comparative transcriptome analysis of the resistance mechanism of; Hemerocallis citrina; Baroni to; Puccinia hemerocallidis; infection. J Plant Interact. 18(1):1–12. doi:10.1080/17429145.2023.2260410.

