# Supplementary figures and images for: The characteristics and analysis of the complete chloroplast genome of Hemerocallis cultivar Small orange lamp 2019 (Asphodelaceae)
Source: Mitochondrial DNA B Resour. 2024 Dec 2;9(12):1632–5. doi: 10.1080/23802359.2024.2435904 (PMC11613412; doi:10.1080/23802359.2024.2435904)

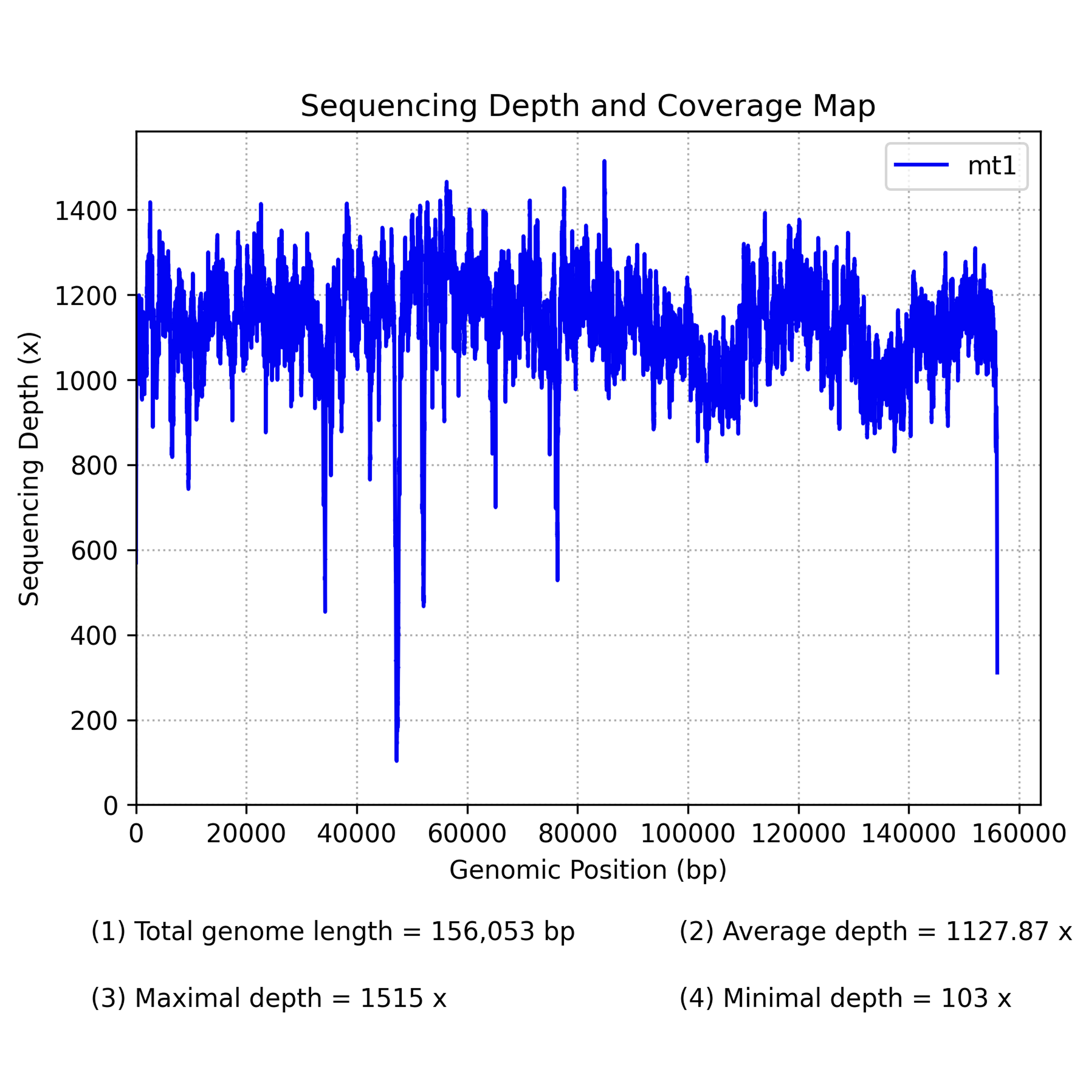

Supplement: Supplemental Material [file TMDN_A_2435904_SM6257.jpg]

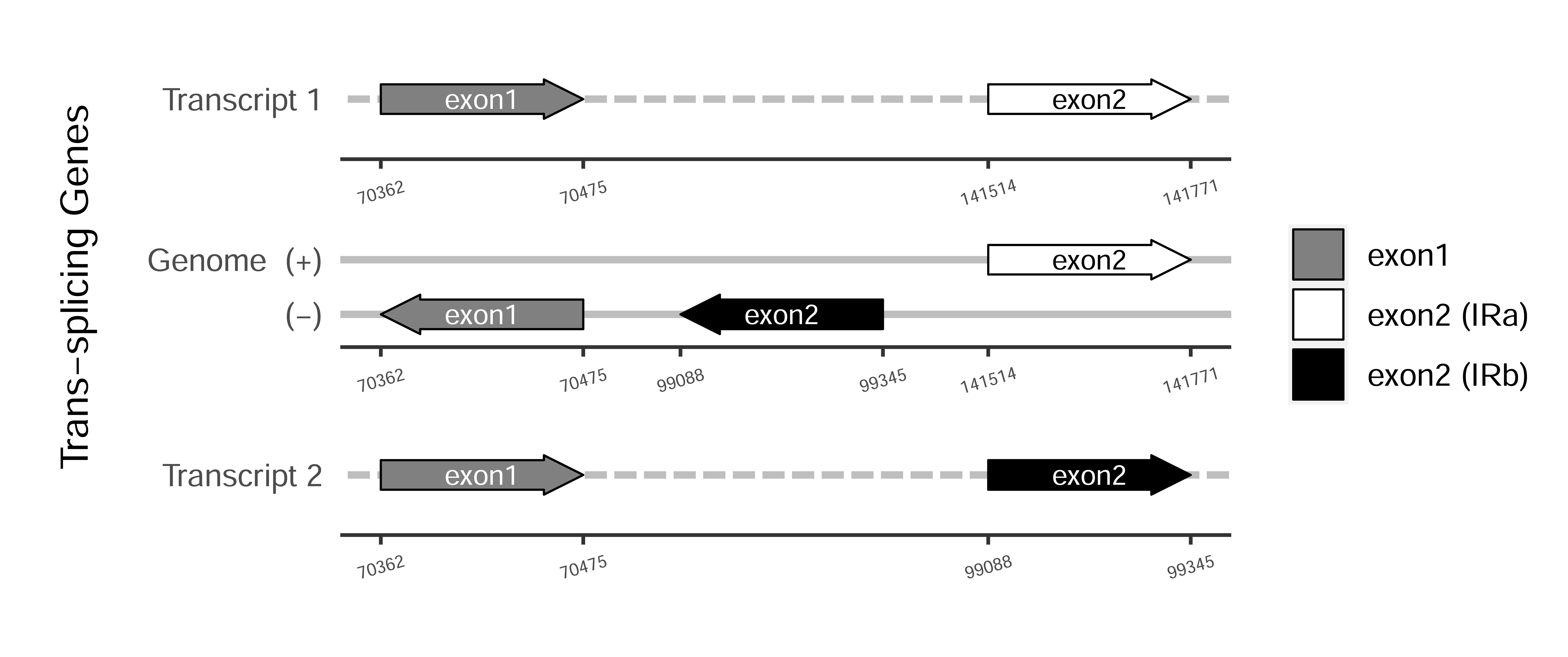

Supplement: Supplemental Material [file TMDN_A_2435904_SM6255.jpg]

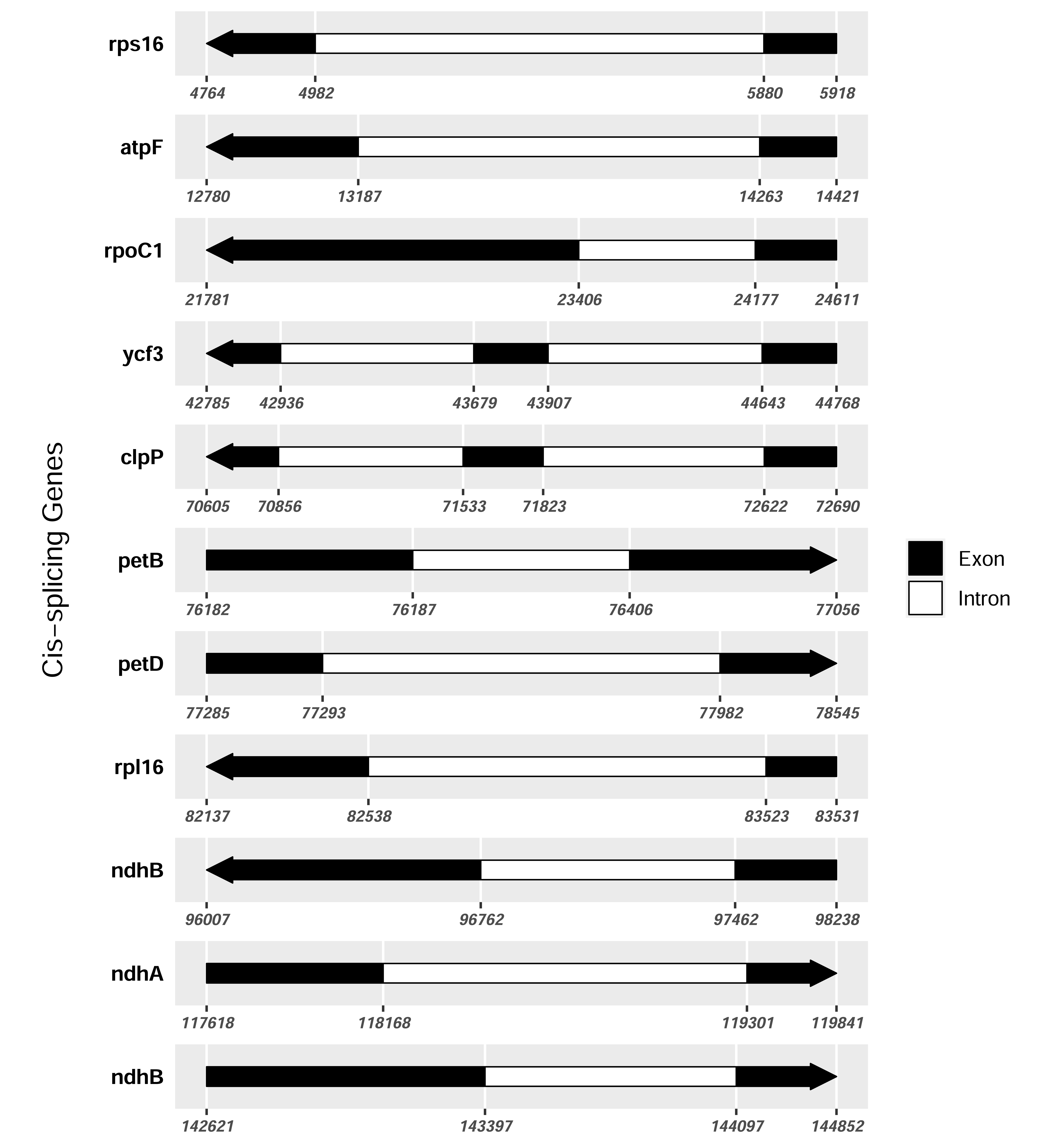

Supplement: Supplemental Material [file TMDN_A_2435904_SM6254.jpg]
